# A Dangerous Partnership: Malignancy and Antiphospholipid Antibodies

**DOI:** 10.5152/eurasianjmed.2026.261434

**Published:** 2026-07-28

**Authors:** Jozélio Freire de Carvalho, Lara Ponte Amadei, Carlos Ewerton Maia Rodrigues

**Affiliations:** 1 Post-graduate Department, Institute of Health Sciences,Federal University of Bahia, Salvador, Brazil; 2 Department of Internal Medicine, Universidade de Fortaleza (Unifor), Fortaleza, Brazil; 3 Graduate Program in Medical Sciences, University of Fortaleza, Fortaleza, Brazil; 4 Department of Internal Medicine, Federal University of Ceará, Fortaleza, Brazil; 5 Department of Internal Medicine, Hospital Geral de Fortaleza, Fortaleza, Brazil

**Keywords:** Antibodies, antiphospholipid, antiphospholipid syndrome, catastrophic antiphospholipid syndrome, neoplasms, paraneoplastic syndromes, thrombosis

## Abstract

Antiphospholipid syndrome (APS) is characterized by persistent antiphospholipid antibodies (aPL) associated with thrombotic and/or obstetric complications. Malignancies are strongly prothrombotic, and growing evidence suggests clinically relevant interactions between aPL and cancer. Interpretation remains challenging because many oncologic studies rely on non-criteria aPL profiles, low titers, or single-time measurements that may not reflect clinically meaningful APS. The aim was to summarize current evidence regarding aPL in malignancy, focusing on prevalence, laboratory profiles, thrombotic manifestations, catastrophic antiphospholipid syndrome (CAPS), paraneoplastic associations, and potential clinical implications in oncology patients. A focused narrative review was conducted using PubMed/MEDLINE and PubMed Central (through October 26, 2025). Predefined search strategies emphasized antibody profile, persistence (≥12 weeks when available), tumor type, and thrombotic outcomes. Studies were selected for clinical relevance and verifiability, with structured synthesis of key findings. Meta-analytic data demonstrate increased anticardiolipin positivity in gastrointestinal, genitourinary, and lung cancers, whereas lupus anticoagulant and anti-β2GPI findings remain heterogeneous. Antiphospholipid antibodies positivity was frequently transient or low titer, limiting clinical interpretation. Persistent or high-risk aPL profiles were associated with increased thrombosis in selected subgroups. Catastrophic antiphospholipid syndrome and paraneoplastic APS have been reported in malignancy, and obstetric APS cohorts suggest a possible bidirectional relationship with cancer incidence. In conclusion, the malignancy–aPL interface involves increased antibody prevalence, phenotype-dependent thrombotic risk, CAPS association, and paraneoplastic mechanisms. Clinical interpretation must consider antibody profile and persistence, avoiding conclusions based on isolated positivity. Prospective studies are needed to clarify clinical relevance and guide screening strategies.

**Main Points**

- Antiphospholipid antibodies (aPL) may be more frequently detected in selected solid tumors—particularly gastrointestinal, genitourinary, and lung cancers—although positivity is often transient or low titer, limiting definitive clinical interpretation.
- Persistent or high-risk aPL profiles, especially lupus anticoagulant positivity, may identify a subset of oncology patients with increased arterial and venous thrombotic risk; however, current evidence does not support routine screening in unselected cancer populations.
- Malignancy may act as a trigger for catastrophic antiphospholipid syndrome, particularly in hematologic cancers, and available registry data suggest poorer outcomes in these patients.
- In selected cases, aPL positivity and thrombotic manifestations may represent a paraneoplastic phenomenon, with antibody disappearance and clinical improvement reported after successful tumor treatment.
- Interpretation of aPL in oncology should remain phenotype-driven and consider antibody profile, titer, persistence, and the multiple prothrombotic mechanisms associated with active malignancy.

## Introduction

Antiphospholipid syndrome (APS) is an acquired autoimmune disorder characterized by the persistent presence of antiphospholipid antibodies (aPL), including lupus anticoagulant (LA), anticardiolipin (aCL), and anti-β2-glycoprotein I (anti-β2GPI), associated with arterial and venous thrombosis and/or obstetric morbidity.^1^ Updates in classification, notably the 2023 ACR/EULAR criteria, have refined diagnostic performance by weighting laboratory domains and clinical manifestations while reinforcing the requirement for aPL persistence in heterogeneous presentations.^2^^,^^3^

Malignancy represents a major prothrombotic condition driven by tumor-expressed tissue factor, inflammatory cytokine release, microparticle shedding, vascular compression, and thrombogenic effects of cancer therapies.^4^ Thromboembolism may be the initial manifestation of cancer, and therefore the coexistence of cancer and aPL becomes clinically relevant due to potentially synergistic mechanisms amplifying thrombosis risk.

Recent evidence demonstrates increased prevalence of aPL in patients with some solid tumors, especially gastrointestinal, genitourinary, and lung cancers, as confirmed by a large meta-analysis of observational studies.^4^ In addition, some cohort studies suggest that cancer patients with aPL positivity may have increased thrombotic risk compared with aPL-negative patients.^5^ Severe APS phenotypes, such as catastrophic antiphospholipid syndrome (CAPS), may be triggered by underlying malignancy, particularly hematologic cancers.^6^ Conversely, cohort data indicate that patients with APS, including those with obstetric APS, have an increased incidence of subsequent malignancy during follow-up.^7^ Moreover, resolution of aPL after successful cancer treatment has been documented, supporting a paraneoplastic mechanism in selected cases.^5^^,^^8^

Given the biological plausibility and clinical implications of this association, better understanding of the cancer-aPL interface is required to guide diagnostic suspicion, risk stratification, and multidisciplinary management.

The objective of this narrative review is to summarize available evidence regarding the relationship between malignancy and aPL, with emphasis on prevalence across different cancers, thrombotic manifestations, catastrophic APS, paraneoplastic associations, and potential clinical implications for risk stratification and multidisciplinary management.

## Methods

A focused narrative review was conducted, prioritizing high-quality syntheses and original studies with public traceability. To improve methodological transparency, search strategies were predefined and conducted using PubMed/MEDLINE and PubMed Central databases (through October 26, 2025), combining controlled terms and keywords including “antiphospholipid,” “anticardiolipin,” “anti-β2-glycoprotein I,” “lupus anticoagulant,” “cancer,” “neoplasm,” “malignancy,” “catastrophic antiphospholipid syndrome,” and “paraneoplastic.”

Studies were considered eligible when they addressed clinical prevalence, thrombotic outcomes, laboratory interpretation, catastrophic APS, or paraneoplastic associations involving aPL in malignancy. Meta-analyses, prospective cohorts, registry-based analyses, and clinically oriented reviews with verifiable data sources were prioritized. Case reports were used selectively to illustrate rare presentations such as malignancy-associated CAPS or paraneoplastic APS.

Exclusion criteria included studies lacking laboratory characterization of aPL profiles, editorials without original data, and reports relying exclusively on isolated non-criteria antibodies without associated thrombotic manifestations, APS-related clinical features, or relevant oncologic correlation. Study selection and data extraction were performed by a single senior reviewer, with emphasis on antibody profile (LA, aCL, anti-β2GPI), titer level, persistence (≥12 weeks when available), tumor type, and thrombotic outcomes.

The most comprehensive meta-analysis on aPL in solid tumors,^4^ clinical reviews and cohorts with thrombotic outcomes,^5^^-^^11^ classical series and registries of CAPS,^12^^-^^15^ studies on specific tumor types (e.g., lung cancer),^9^^,^^16^ and publications addressing criteria and laboratory fundamentals were prioritized.^1^^-^^3^^,^^17^ Although this work follows a narrative design rather than a systematic review, methodological transparency was reinforced by explicit selection criteria and structured synthesis of the evidence to enhance clinical interpretability.

Because this narrative review was not designed as a formal systematic review, study selection and data extraction were performed by a single senior reviewer. This approach may increase the risk of selection bias and should be interpreted as a methodological limitation.

This study used ChatGPT (OpenAI) to improve language, edit grammar, and enhance readability during the preparation of the manuscript.

### Definition and Interpretation of Antiphospholipid Antibody Positivity

Because interpretation of aPL results is highly context-dependent, particular attention was paid to how aPL positivity was defined across the included studies. Many oncology studies assessed aPL using single-time measurements, low titers, or non-criteria profiles (e.g., isolated Immunoglobulin M (IgM) aCL), without systematic confirmation of persistence. Such findings were interpreted with caution, as they do not fulfill laboratory criteria for APS and may reflect transient immune activation, inflammation, or paraneoplastic phenomena rather than clinically meaningful APS. When available, information on antibody isotype, titer, and persistence (repeat testing ≥12 weeks) was extracted and emphasized, and greater clinical relevance was attributed to persistent, high-titer, or ­LA–­positive profiles, particularly when associated with thrombotic manifestations.

### BOX—Operational Interpretation of Antiphospholipid Antibodies in Malignancy

- Criterion aPL: LA, aCL Immunoglobulin G (IgG)/IgM (moderate–high titer), anti-β2GPI IgG/IgM
- Non-criterion: isolated low-titer antibodies or non-standard assays
- High-risk profile: LA positivity, triple positivity, persistent ≥12 weeks
- Lower clinical significance: single-time positivity, isolated IgM, low titers

Interpretation must consider tumor-related prothrombotic mechanisms.

## Results

### Prevalence of Antiphospholipid Antibodies in Patients with Malignancy

The most robust evidence regarding the prevalence of aPL in malignancy derives from the systematic review and meta-analysis by Abdel-Wahab et al, which aggregated data from 33 observational studies of solid tumors.^4^ This analysis demonstrated a significantly higher probability of aCL positivity in gastrointestinal cancers (relative risk [RR] 5.1; 95% CI: 2.6-9.95), genitourinary malignancies (RR 7.3; 95% CI 3.3-16.2), and lung cancer (RR 5.2; 95% CI 1.3-20.6) compared with controls. In contrast, results for LA and anti-β2-glycoprotein I (anti-β2GPI) antibodies were heterogeneous and inconsistent across studies, largely reflecting variability in laboratory assays, cut-off values, and study design.^4^

Earlier narrative reviews and cohort studies similarly reported increased aPL prevalence in oncologic populations, particularly in solid and hematologic malignancies, but emphasized important methodological limitations, including single-time testing, low antibody titers, lack of standardized assays, and limited evaluation of antibody persistence.^5^^,^^6^ Studies focusing on solid tumors complicated by venous thromboembolism (VTE) found that aPL positivity was generally infrequent and often transient, suggesting limited utility of routine aPL screening in unselected cancer populations.^7^^,^^8^ Overall, while aPL positivity appears more frequent in certain malignancies than in the general population, its magnitude and clinical relevance vary substantially according to antibody profile, titer, persistence, and tumor type.^4^^,^^5^ See Table 1 for a summary of these studies.

### Thrombotic Risk Associated with Antiphospholipid Antibodies in the Oncologic Setting

Malignancy is a strong independent risk factor for thrombosis, and the presence of aPL may further modulate thrombotic risk in selected patient subsets. In the aforementioned meta-analysis, a subgroup analysis of lung cancer patients showed an approximately 3.8-fold increased risk of thrombotic events among aPL-positive individuals compared with aPL-negative counterparts.^4^ These findings were supported by a subsequent prospective cohort study demonstrating that ambulatory cancer patients with aPL positivity had an approximately 3.6-fold higher risk of combined arterial and venous thrombotic events, particularly when LA or aCL (IgG/IgM) were present.^9^

Conversely, other studies reported a low prevalence of aPL and frequent antibody transience in patients with cancer-associated VTE, casting doubt on a direct pathogenic role of aPL in many cases of malignancy-related thrombosis.^7^^,^^8^ Taken together, available evidence suggests that persistent aPL positivity—especially LA positivity or high-risk laboratory profiles—may identify a subgroup of oncology patients with heightened thrombotic risk, whereas isolated or transient aPL findings are often of uncertain clinical significance. Importantly, current data do not support universal aPL screening or systematic modification of anticoagulation strategies based solely on aPL positivity in patients with cancer.^7^^-^^9^

### Catastrophic Antiphospholipid Syndrome and Malignancy

Catastrophic antiphospholipid syndrome is a rare but severe manifestation of APS characterized by rapidly progressive multiorgan thrombosis. Data from classical case series and registry analyses indicate that a clinically relevant proportion of CAPS cases occur in association with malignancy, particularly hematologic cancers such as lymphoma and leukemia.^12^^-^^15^ In older series, malignancy was reported in approximately 9% of CAPS cases, often acting as a precipitating “second-hit.”^12^^,^^13^

Several studies have suggested that CAPS occurring in the context of active malignancy is associated with worse outcomes and lower recovery rates compared with CAPS unrelated to cancer.^13^^-^^15^ These observations highlight the importance of considering occult or active malignancy in patients presenting with explosive or atypical APS manifestations and emphasize that optimal management of malignancy-associated CAPS requires not only standard APS-directed therapy but also control of the underlying tumor when feasible.

### Antiphospholipid Syndrome as a Paraneoplastic Phenomenon

Antiphospholipid syndrome and aPL positivity may represent a paraneoplastic phenomenon in selected patients. Clinical series and case-based evidence describe new-onset aPL positivity and thrombotic manifestations occurring in temporal association with malignancy, with remission of aPL and thrombosis following successful tumor treatment in some cases.^5^^,^^10^^,^^11^ In a large ­clinical–pathologic series, malignancy-­associated aPL were linked to venous and arterial thrombosis, thrombocytopenia, and, in a subset of patients, CAPS triggered by the underlying neoplasm.^10^

These observations support the concept that, particularly in late-onset APS, atypical clinical courses, or newly detected aPL profiles, an underlying malignancy should be considered. Disappearance of aPL after effective oncologic therapy is a strong indicator of a paraneoplastic relationship in selected cases.^5^^,^^10^^,^^11^

### Tumor-Specific Features

The association between malignancy and aPL/APS varies across tumor types. In solid tumors, the meta-analysis by Abdel-Wahab et al identified gastrointestinal, genitourinary, and lung cancers as those most consistently associated with increased aCL positivity.^4^ In lung cancer, some observational studies have reported associations between LA positivity and increased thrombotic risk, particularly in non-small-cell lung cancer; however, results remain heterogeneous and should be interpreted cautiously due to differences in study design, laboratory methodology, and patient populations.^9^^,^^16^

In hematologic malignancies, aPL positivity has been described in subsets of patients with aggressive lymphomas and leukemias. Although some studies suggested possible prognostic implications, current findings remain inconsistent and insufficient to establish definitive conclusions regarding prognosis or causality.^6^^,^^10^^,^^11^ By contrast, in unselected solid tumors complicated by VTE, routine aPL testing does not appear to improve thrombotic risk prediction, as antibody positivity is often transient and of low titer.^7^^,^^8^ These findings underscore the importance of considering tumor type, disease stage, antineoplastic therapies, and detailed aPL profiles when interpreting results.

### Bidirectional Relationship Between Malignancy and Antiphospholipid Antibodies/Antiphospholipid Syndrome

Available evidence suggests a bidirectional relationship between malignancy and aPL/APS. On one hand, increased aPL prevalence and higher thrombotic risk have been documented in selected cancer subgroups, particularly lung cancer.^4^^,^^9^^,^^16^ On the other hand, cohort studies in obstetric APS have demonstrated an increased incidence of subsequent malignancy during ­follow-up, with LA positivity associated with incident cancer.^6^

Although causality cannot be established from available data, these observations raise the possibility that malignancy may promote aPL generation through immune dysregulation or paraneoplastic mechanisms, while APS-related chronic inflammation or immune activation may contribute to cancer risk in selected patients.^4^^-^^6^ This bidirectional association has important implications for clinical vigilance and multidisciplinary management.

## Discussion

**Figure 1.**
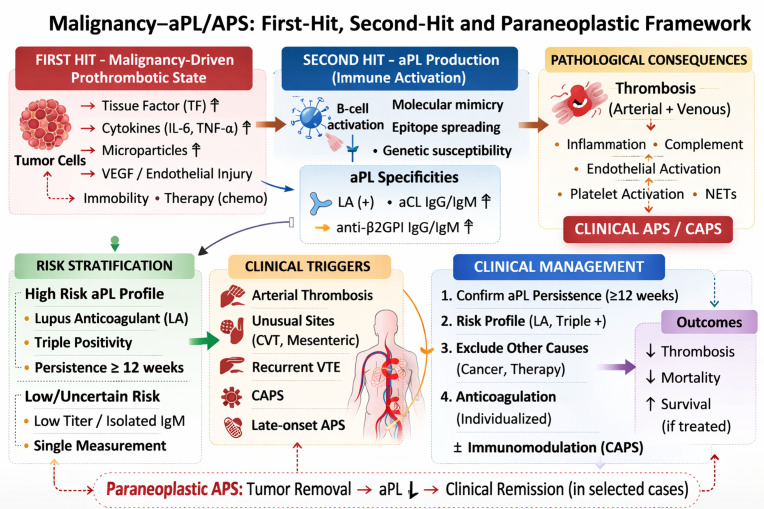
Malignancy–aPL/APS interface: first-hit/second-hit and paraneoplastic framework linking cancer-related thrombosis and antiphospholipid autoimmunity. Conceptual diagram illustrating the bidirectional interaction between malignancy and aPL. The “first-hit” represents malignancy-driven prothrombotic and inflammatory pathways, including tissue factor expression, cytokine release, endothelial injury, and therapy-related triggers. The “second-hit” depicts immune activation leading to aPL production through mechanisms such as molecular mimicry, epitope spreading, and paraneoplastic B-cell activation. Downstream effects include endothelial dysfunction, complement activation, platelet activation, and amplification of thrombosis, potentially contributing to venous and arterial thrombotic events or CAPS. Risk stratification is influenced by antibody profile, persistence, and LA positivity. In selected cases, tumor control may lead to reduction of aPL levels and clinical remission, supporting a paraneoplastic model. aCL, anticardiolipin antibodies; anti-β2GPI, anti-β2-glycoprotein I antibodies; aPL, antiphospholipid antibodies; APS, antiphospholipid syndrome; CAPS, catastrophic antiphospholipid syndrome; LA, lupus anticoagulant; NETs, neutrophil extracellular traps; TF, tissue factor; VEGF, vascular endothelial growth factor; VTE, venous thromboembolism.

This narrative review highlights a complex and clinically relevant interface between malignancy and aPL, spanning increased aPL prevalence in selected cancers, modulation of thrombotic risk, CAPS triggered by malignancy, and paraneoplastic phenomena. However, careful interpretation is essential, as much of the available literature is limited by heterogeneity in tumor types, laboratory definitions, and lack of standardized assessment of aPL persistence. A conceptual overview of these interacting mechanisms is illustrated in Figure 1, integrating malignancy-driven thrombosis, immune activation, and paraneoplastic pathways.

Importantly, classification criteria should not replace clinical judgment in oncology patients with thrombosis, and interpretation of aPL must remain phenotype-driven and context-specific.

A central challenge in this field is the definition and interpretation of aPL positivity in oncology. Many studies reporting increased aPL prevalence in cancer rely on single-time measurements, low titers, or non-criteria profiles, particularly isolated IgM aCL antibodies.^4^^,^^5^^,^^7^^,^^8^ According to international consensus and updated ACR/EULAR classification criteria, APS requires persistent aPL positivity, defined by repeat testing at least 12 weeks apart, using standardized assays for LA, aCL, and anti-β2-glycoprotein I antibodies.^1^^-^^3^^,^^17^ Failure to apply these criteria substantially limits the clinical relevance of many oncology studies and raises the possibility that part of the reported aPL positivity reflects transient immune activation, inflammation, or paraneoplastic epiphenomena rather than bona fide APS.

From a clinical perspective, available data suggest that not all aPL profiles carry equivalent thrombotic risk in patients with malignancy. While several studies indicate that persistent or high-risk laboratory profiles—particularly LA ­positivity—are associated with increased arterial and venous thrombosis in selected oncologic subgroups, such as lung cancer, other investigations demonstrate low prevalence and frequent transience of aPL in unselected cancer-­associated venous thromboembolism.^4^^,^^7^^-^^9^^,^^16^ These findings argue against routine aPL screening in all patients with malignancy and instead support a targeted approach focused on patients with unexplained, recurrent, or atypical thrombotic events, especially arterial thrombosis or thrombosis at unusual sites.

The interaction between malignancy and APS classification warrants particular caution. Active cancer represents a strong prothrombotic and proinflammatory state that may confound APS classification by contributing to thrombosis independently of aPL. Moreover, cancer-related ­factors—including chemotherapy, central venous catheters, infection, immobility, and endothelial injury—may act as alternative explanations for thrombotic events, complicating attribution to APS. As a result, applying ACR/EULAR APS classification criteria in the setting of active malignancy requires careful clinical judgment, and classification should not be equated with diagnosis in individual patients.^1^^-^^3^ This is particularly relevant in late-onset APS, CAPS, or atypical presentations, where malignancy should be actively considered as a potential trigger or underlying condition.

Catastrophic antiphospholipid syndrome represents an extreme intersection between APS and malignancy. Registry data and classical series consistently show that malignancy, especially hematologic cancers, can act as a precipitating “second-hit” for CAPS and is associated with worse outcomes.^12^^-^^15^ In such cases, optimal management extends beyond standard CAPS therapy—including anticoagulation, high-dose corticosteroids, and plasma exchange or intravenous immunoglobulin—to encompass prompt identification and treatment of the underlying malignancy. Failure to control the neoplasm may limit therapeutic response and worsen prognosis.

Paraneoplastic APS further illustrates the bidirectional nature of this relationship. Numerous reports document new-onset aPL positivity and thrombotic manifestations temporally linked to malignancy, with resolution of antibodies and clinical events following successful cancer treatment.^5^^,^^10^^,^^11^ These observations reinforce the need to consider occult malignancy in patients presenting with late-onset APS, rapidly progressive disease, or unusual serologic patterns, and support the concept that, in selected cases, aPL production may be driven by tumor-related immune dysregulation.

Conversely, cohort data indicate that patients with APS—particularly obstetric APS—may have an increased incidence of subsequent malignancy during long-term follow-up, with LA positivity emerging as a potential risk marker.^6^ Although causality remains unproven, this finding raises important questions regarding chronic immune activation, inflammation, and shared pathogenic pathways linking APS and cancer. At present, these data support heightened clinical vigilance rather than systematic oncologic screening in all APS patients.

Taken together, the available evidence supports a nuanced and phenotype-driven interpretation of aPL in malignancy. Antibody profile, titer, and persistence—as emphasized by ACR/EULAR criteria—are critical determinants of clinical relevance and should guide interpretation, risk stratification, and management. Until robust prospective studies with standardized laboratory definitions become available, individualized and multidisciplinary decision-making remains essential in patients at the intersection of cancer and aPL/APS.

### Pathogenic Mechanisms Proposed

The interaction between malignancy and aPL/APS can be understood in a “first-hit/second-hit” model: aPL provide a basal prothrombotic state, while the tumor microenvironment—with tissue factor, microparticles, endothelial/platelet activation, and inflammation—acts as a trigger for thrombotic events and, rarely, for CAPS.^3^^-^^5^^,^^13^^-^^15^^,^^17^ From an immuno-molecular standpoint, anti-β2GPI may bind endothelial/platelet targets, amplifying coagulation and adhesion; tumors may expose altered ­phospholipid-associated antigens and break tolerance, inducing aPL production. The transience of aPL in some oncologic settings and negative conversion after tumor treatment support a paraneoplastic link in selected patients.^5^^,^^7^^,^^8^^,^^10^^,^^11^

To enhance conceptual clarity, a first-hit/second-hit framework integrating tumor-related triggers and paraneoplastic immune activation is proposed.

### Suggested Immunological Investigations and Therapeutic Considerations

In selected oncology patients with persistent or clinically significant aPL positivity, additional immunological evaluation may help refine thrombotic risk assessment and improve clinical interpretation. Potential investigations include expanded aPL profiling (including persistence confirmation), LA functional testing, complement activation markers, non-criteria aPL antibodies, cytokine profiling, and immune-cell phenotyping in selected research settings. However, the clinical utility of these investigations remains incompletely established and requires prospective validation.

Regarding treatment response, current evidence remains limited and derives predominantly from observational studies, CAPS registries, and case-based reports. Standard management generally includes anticoagulation according to thrombotic phenotype and overall ­oncologic risk. In CAPS associated with malignancy, treatment typically combines anticoagulation, corticosteroids, plasma exchange and/or intravenous immunoglobulin, together with aggressive treatment of the underlying neoplasm.^12^^-^^15^ Data regarding immunosuppressive biologics or targeted therapies in malignancy-associated APS remain scarce, and no standardized therapeutic recommendations can currently be established.

Available reports suggest variable clinical responses, with some patients improving after combined anticoagulation and tumor-directed therapy, whereas outcomes in malignancy-­associated CAPS remain poor in severe presentations.

### Clinical Implications

Clinical algorithm—When to test for aPL in cancer

Consider testing when:

- arterial thrombosis,
- unusual site thrombosis,
- recurrence despite anticoagulation,
- CAPS suspicion, and
- late-onset APS phenotype.

If positive:

- Confirm persistence ≥12 weeks,
- Define laboratory risk profile,
- Evaluate malignancy-related confounders, and
- Individualize anticoagulation strategy.

For oncologists/hematologists/rheumatologists, several practical principles emerge. In cancer patients with unexplained/recurrent ­thrombosis—especially arterial or at unusual sites—testing for aPL may help risk stratification, ideally with repeat testing after ≥12 weeks if APS classification is intended.^1^^-^^3^ In late-onset APS, CAPS, or atypical courses, hidden malignancy should be considered. For anticoagulation strategies, no specific recommendations exist based solely on aPL positivity; decisions should integrate global risk (tumor type, therapy, thrombosis history, bleeding risk). In CAPS associated with malignancy, tumor control is an integral part of treatment alongside anticoagulation and immunomodulation.^13^^-^^15^

### Limitations of the Evidence

The available evidence is predominantly based on observational studies, registry analyses, and case series, with substantial heterogeneity regarding tumor type, laboratory methodology, antibody profile, and timing of aPL assessment.^4^^,^^7^^-^^9^ Many studies relied on single-time antibody measurements without confirmation of persistence ≥12 weeks, limiting interpretation according to current APS classification criteria.^1^^-^^3^ In addition, oncologic confounders such as chemotherapy, infections, central venous catheters, and tumor-related thrombogenicity complicate attribution of thrombotic events directly to aPL-mediated mechanisms. Finally, prospective studies evaluating standardized screening strategies, long-term outcomes, and aPL-guided management in oncology patients remain limited.

## Conclusion

The available literature supports a clinically relevant association between malignancy and aPL, involving increased antibody prevalence in selected cancers, potential modulation of thrombotic risk, CAPS triggered by malignancy, and paraneoplastic manifestations. However, interpretation of aPL positivity in oncology requires caution, particularly when antibodies are transient, low titer, or assessed only once. Antibody profile, persistence, and clinical phenotype remain critical for interpretation and risk stratification. Current evidence does not support routine aPL screening in unselected cancer populations, underscoring the need for individualized and multidisciplinary clinical assessment. Prospective studies with standardized laboratory definitions and clinically meaningful outcomes are needed to better define the relevance of aPL in malignancy. Until such evidence becomes available, interpretation of aPL in oncology should remain individualized and context-dependent.

## Figures and Tables

**Table 1.** Structured Summary of Major Evidence Linking Malignancy and Antiphospholipid Antibodies/Antiphospholipid Syndrome

Axis	Key Findings	Representative Evidence	Sample Size/Design	Clinical Message	References
aPL prevalence in malignancy	Increased aPL positivity, especially aCL, reported in solid tumors such as gastrointestinal, genitourinary, and lung cancers. Heterogeneity in laboratory methods and transient results are common.	Meta-analysis of observational studies demonstrated higher aCL positivity in solid tumors vs. controls; variable significance for LA and anti-β2GPI.	Meta-analysis of 33 observational studies; narrative syntheses.	aPL positivity is more frequent in selected cancers but often transient and of uncertain clinical significance.	^4^^,^^5^
Thrombotic risk in aPL-positive cancer patients	Persistent or high-risk aPL profiles have been associated with increased arterial and venous thrombotic risk in selected oncologic subgroups.	Lung cancer cohorts and ambulatory cancer patient studies showed increased combined arterial and venous events when aPL are present.	Prospective cohort and subgroup analyses.	High-risk profiles (LA, persistence) may identify patients with increased thrombosis risk; routine screening not supported.	^4^^,^^5^^,^^9^^,^^16^
APS severity and malignancy (CAPS)	Malignancy, especially hematologic cancers, may trigger catastrophic APS and is associated with worse prognosis and reduced recovery.	CAPS registry analyses and classical case series describe malignancy acting as a clinical “second-hit.”	Registry data and historical clinical series.	Consider underlying malignancy in explosive or atypical APS/CAPS presentations.	^12^^-^^15^
Paraneoplastic and bidirectional associations	APS/aPL may reflect a paraneoplastic phenomenon with remission after tumor treatment; APS cohorts show increased cancer incidence during follow-up.	Clinical–pathologic series and obstetric APS cohort data demonstrating temporal association and antibody disappearance after cancer therapy.	Cohort studies and observational series.	Late-onset APS or atypical serology should prompt evaluation for occult malignancy.	^5^^-^^8^^,^^10^^,^^11^

aCL, anticardiolipin antibodies; anti-β2GPI, anti-β2-glycoprotein I antibodies; aPL, antiphospholipid antibodies; APS, antiphospholipid syndrome; CAPS, catastrophic antiphospholipid syndrome; LA, lupus anticoagulant; VTE, venous thromboembolism.

## Data Availability

The data that support the findings of this study are available on request from the corresponding author.
